# Circular RNAs as New Regulators in Gastric Cancer: Diagnosis and Cancer Therapy

**DOI:** 10.3389/fonc.2020.01526

**Published:** 2020-09-18

**Authors:** Wei Wu, Tianyuan Zhen, Junmin Yu, Qingli Yang

**Affiliations:** ^1^College of Food Science and Engineering, Qingdao Agricultural University, Qingdao, China; ^2^Institute for Translational Medicine, Qingdao University, Qingdao, China; ^3^Department of Anesthesiology, The Affiliated Hospital of Qingdao University, Qingdao University, Qingdao, China

**Keywords:** gastric cancer, circRNA, chemotherapy, cell growth, cell cycle

## Abstract

Gastric cancer (GC) is one of the most commonly diagnosed cancers that causes high mortality in the world. Although the surgery tools and chemotherapies have significantly improved the overall survival of patients with GC, the early diagnosis of GC remains insufficient and many patients diagnosed with advanced stages of GC are not able to benefit from curative therapy. Circular RNAs (circRNAs), novel members of the non-coding cancer genome, are being explored with regards to various cancer types including GC. CircRNAs could work as miRNA sponges to regulate cell proliferation, cell migration, and cell cycle in GC. In addition, it was found that abnormal expression of circRNAs was associated with pathological characteristics in GC tissues, which could help to act as potential markers of early diagnosis or predictors of prognosis. Although various functional circRNAs have been discovered and characterized, the studies of circRNAs in GC are still at early stages compared with other RNAs. In order to provide a whole view to better understand the circRNAs in the occurrence and development of GC, we review the current knowledge on circRNAs in relation to their expression and regulation in GC as well as their potential to be diagnosis markers, and their role in drug resistance will be mentioned. It is helpful to address their possibility from basic research into practical application.

## Background

### Gastric Cancer

Gastric cancer (GC) is one of the most commonly diagnosed cancers. The mortality of GC accounts for 8.2% of all cancer death, being the third leading cause of cancer-related death in the world. Eastern Asia is well known to be a high incidence area of GC, the incident rates are markedly elevated especially in Mongolia, Japan, and the South Korea (1). Anatomically, GC could be classified into true gastric (non-cardia) and gastro-oesophageal-junction cancers (cardia). Through genomic and statistical analysis GC could be classified into four subgroups: Epstein-Barr virus positive tumors (9%), microsatellite unstable tumors (22%), genomically stable tumors (20%), and chromosomally unstable tumors (50%), which might be used in the determination of prognosis and treatment (2). The important causes of GC include Helicobacter pylori infection and Epstein-Barr virus (EBV) infection, and 10% of the cases are aggregated in families (3).

Most patients with early stage GC cannot be diagnosed because it begins as asymptomatic, and, when they are diagnosed with symptoms of anorexia, dyspepsia, weight loss and abnormal pain, often it is at an advanced stage, with an overall survival (OS) rate of less than 30% (4). Despite the improvement of surgical and medical managements, it is still a big challenge to completely eliminate tumor cells via surgery, radio therapies and chemotherapies. In this scenario, it is essential to find new potential diagnosis biomarkers and therapeutic targets to improve the diagnosis and treatment of GC. In recent years, the functions of circRNAs in GC have been significantly evaluated since their discovery 40 years ago. In this review, the biogenetic process and biological functions of circRNAs, and their close relationship with GC are summarized, and the clinical significance of circRNAs with regulatory potency in the diagnosis and treatment of GC are emphasized.

### CircRNAs

Non-coding RNAs (ncRNA) represent 60% of the total RNA by genome wide analysis. According to their functions, ncRNA can be classified into house-keeping ncRNAs and regulatory ncRNAs. House-keeping ncRNAs include, ribosomal RNA (rRNA), transfer RNAs (tRNA), small nuclear RNA (snRNA)and small nucleolar RNA (snoRNA), which participate in mRNA and rRNA processing. Regulatory ncRNAs are composed of microRNAs (miRNAs), siRNAs, piwi-interacting RNAs (piRNAs) and others (<200 bp) and long ncRNAs (lncRNAs) (>200 bp), which show their ability to control gene expression as epigenetic regulators. Among lncRNAs, circular RNAs (circRNAs) featured in high abundance, structural stability, and tissue- and developmental-specific expression, which have recently been regarded as an emerging key player in regulating various physiological processes and pathogenic disease progress (5). Recent studies indicated that circRNAs are crucial in several types of diseases, which include neurological disorders (6), atherosclerosis (7, 8), osteoarthritis (9), diabetes (10) and cancer (11). Based on the increased evidences of the significance of circRNAs in human diseases, circRNAs may act as a diagnosis biomarker and a new therapeutic target in the near future.

**FIGURE 1:**
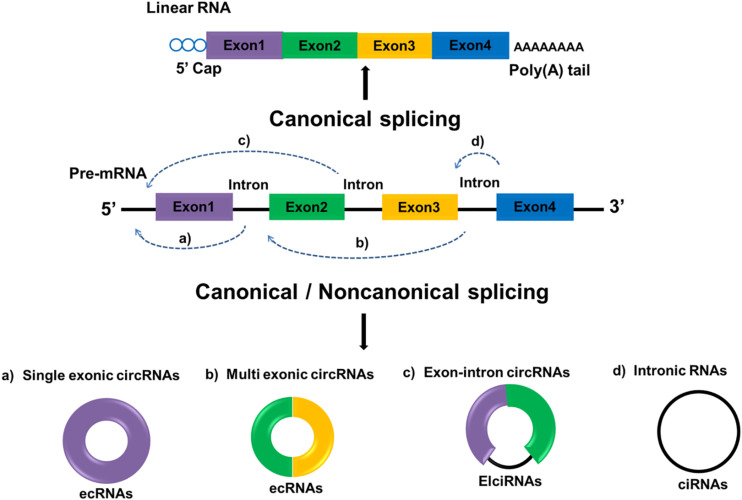
Biogenesis of circRNA. Linear mRNAs are produced by canonical splicing while circRNAs can be produced through both canonical and non-canonical splicing. Exon-derived circRNA (ecRNA) generates from single exons **(a)** or multiple exons **(b)**. Exon–intron circRNA (EIciRNA) consists of two exons between which an intron is inserted **(c)**. Circular intronic RNA (ciRNA) generates from only introns **(d)**. Rectangles in different colors represent exons and black lines represent introns.

Circular RNAs are defined as a distinct subclass of endogenous lncRNAs which form a locked continuous loop covalently with the 5′ and 3′ ends joined together, transcribed through RNA polymerase II as most lncRNAs are. Through canonical and non-canonical splicing, circRNAs can be categorized into three types: exonic circRNA (ecRNAs), originated from pre-mRNAs consisting of single or multiple exons; circular intronic RNAs (ciRNAs), derived from linear RNAs with one or more introns; and those composed of both exons and introns are named exon-intron circRNAs (ElciRNAs) (Figure 1). CircRNAs are highly valued because of their distinctive roles in gene expression regulation and biological process (12). Regulatory roles in gene by circRNAs can be classified as follows: (1) CircRNAs can bind to the promoter of target genes as well as RNA polymerase II to regulate gene transcription; (2) CircRNAs can have different gene splicing patterns by sequestering RBPs that regulate mRNA processing; (3) CircRNAs can compete endogenous miRNA-binding sites by sponging miRNAs in target genes; and (4) CircRNAs can be translated into proteins. Although the functions and molecular mechanism of circRNAs remains largely unexplored, recent evidences suggested that circRNAs are aberrant expressed in several cancer types with tissue-and disease-specificity, making them a potential diagnosis biomarker in cancer (13). For instance, the expression of circRNAs can change in many cancers including, prostate cancer (14), bladder cancer (15), breast cancer (16) and glioma (17). A large number of researches also indicated that circRNAs evaluation in cancer patients may be helpful for prognosis prediction of certain cancers. For instance, compared with low circ-FBXW7 expression in glioblastoma patients, higher circ-FBXW7 expression exhibited higher overall survival (OS) (18). The down-regulation of circHIPK3 in bladder cancer was closely related to increased invasion as well as lymph node metastasis (19). Hereinafter we emphasize the expression profiles of circRNAs and circRNAs-associated therapeutic potentials in GC.

### CircRNAs and Gastric Cancer

As significant attention has been received in recent years regarding the role of circRNAs in GC diagnosis and treatment, many papers report high-throughput sequencing or microarray results that describes the differential circRNAs expression profiles in GC patients and healthy donors (20, 21). These identified circRNAs are down-regulated or up-regulated during carcinogenesis, and therefore serve as tumor suppressors or oncogenes in GC, which make possible that circRNAs become diagnosis biomarkers and prognosis predictors. We searched recent published articles in key journals, from which the expressions of circRNAs and their clinical significances associated with human GC are summarized below (Table 1 and Figure 1).

**TABLE 1:** Expression of CircRNAs associated with human gastric cancers.

CircRNAs	Gene symbol	Deregulation	References	Gene affected	Function affected
circPVT1	*PVT1*	Increased	(22)	miR-125	Cell growth
circLMTK2	*LMTK2*	Increased	(23)		Cell growth and prognosis
hsa_circ_0000096	*HIAT1*	Decreased	(24)	Cyclin D1, CDK6, MMP-2, MMP-9	Cell growth and migration
circLARP4	*LARP4*	Decreased	(25)	miR-424, LATS1	Cell growth and invasion
circRNA_100269	*LPHN2*	Decreased	(26)	miR-630	Cell growth
circYAP1	*YAP1*	Decreased	(27)	miR-367-5p	Cell growth and invasion
hsa_circ_0130810	*KIAA1244*	Decreased	(20)		Cell metastasis
hsa_circ_0000993	*ATL2*	Decreased	(28)	miR-214-5p	Cell growth and metastasis
circAGO2	*AGO2*	Increased	(29)		Cell growth, invasion, and metastasis
circ-DONSON	*DONSON*	Increased	(30)	SOX4	Cell growth and invasion
has_circ_0001461	*FAT1*	Decreased	(31)	miR-548g, RUNX1	Cell growth and metastasis
hsa_circ_0006156	*FNDC3B*	Increased	(32)	E-cadherin, CD44	Cell migration and invasion
hsa_circ_0000199	*AKT3*	Increased	(33)	miR-198, PIK3R1	Cell growth and apoptosis
hsa_circ_0064644	*RBMS3*	Increased	(34)	miR-153, SNAI1	Cell growth and invasion
circPDSS1	*PDSS1*	Increased	(35)	miR-186-5p, NEK2	Cell cycle and apoptosis
hsa_circ_0000234	*CUL2*	Decreased	(36)	mir-142-3p, VAMP3	Cell growth and metastasis
circPSMC3	*PSMC3*	Decreased	(37)	miR-296-5p	Cell growth and migration
circNF1	*NF1*	Increased	(38)	miR-16	Cell growth
hsa_circ_0010522	*RAP1GAP*	Increased	(39)	miR-133	White adipose tissue browning, cancer-associated cachexia
has_circ_0032627	*DLST*	Increased	(40)	miR-502-3p, NRAS/MEK1/ERK1/2	Cell viability, invasion, and metastasis
hsa_circ_0092303	*CACTIN*	Increased	(41)	miR-331-3p, TGFBR1	Cell growth and metastasis
circNRIP1	*NRIP1*	Increased	(42)	miR-149-5p	Cell growth and metastasis

#### CircRNA and Gene Expression Profiles in GC

Several articles published in 2017 show us a preliminary expression profile of circRNAs in GC. Huang’s group screened with circRNA chip using five pairs of matched GC and non-GC tissues, and it was revealed that 191 circRNAs were increased in GC tissues, whereas 522 were decreased, among which hsa_circ_0076304, hsa_circ_0076305, and hsa_circ_0035431 were mostly changed (43). Guo’s group detected a total of 5396 circRNAs between GC tissues and adjacent non-tumorous tissues, revealed 107 increased and 201 decreased circRNAs. Among which hsa_circ_0035445, hsa_circ_0003789, hsa_circ_0007099, hsa_circ_0001897, and hsa_circ_0007707 had the highest magnitude of change. Moreover, most differentially expressed circRNAs were located in chr1, chr2, chr3, chr9, and chr17 (44). Dai’s group screened circRNA and mRNA profiles in the GC tissues and adjacent tissues and found 1,285 significant differently expressed circRNAs, with 594 decreased and 691 increased, and 69 among them might play regulatory roles in their target gene mRNAs expression. 5,460 differentially expressed genes were found in 29,112 genes with 2,390 genes increased and 3,070 genes decreased, such as MYH9, MALAT1 may involve in the occurrence and development of GC (45). A Brazil group verified 66 annotated circRNAs in Gastric tissue without GC, 620 in paired tumor-adjacent gastric tissue and 220 in GC samples, among them, hsa_circ_0001136, hsa_circ_0000284, hsa_circ_0000211, and hsa_circ_0004771 were differentially expressed which may become novel biomarkers of GC (46). Guo’s group screened the profiles in circRNAs expression between the plasma of GC patients and healthy donors and the circRNA levels were confirmed by both RT-qPCR and RT-droplet digital PCR. They found 343 differentially expressed circRNAs with hsa_circ_0088300 and hsa_circ_0075825 increased, hsa_circ_0001017 and hsa_circ_0061276 decreased in patients. The plasma hsa_circ_0001017 and hsa_circ_0061276 in patients after operation recovered to normal level indicating that these two circRNAs can be new ideal biomarkers for GC (47). In 2019, Zhang’s group identified 196 increased and 311 decreased genes in GC tissues. In the meantime, they identified two decreased circRNAs hsa_circ_0000332 and hsa_circ_0021087 in GC tissues. It was also found that hsa_circ_0021087 may be more crucial in the GC network than hsa_circ_0000332 through prediction of target genes for circRNAs and miRNAs and construction of differentially expressed circRNA–miRNA–mRNA networks (48).

#### CircRNA Serves as MiRNA Sponges in GC

MicroRNAs are key players in the pathogenesis of almost all human cancer types (49, 50). Previously we mentioned that circRNAs can regulate gene expression by serving as miRNA sponges. CircRNAs have a miRNA response element that can compete for miRNA-binding sites in target genes. Although circRNAs regarding their potential molecular mechanisms in GC are largely unexplored, the function as regulators of miRNA activity suggested their involvement in GC. Recent papers drew some inspiration on the role of circRNAs acting as miRNA sponges in regulating GC cell proliferation, invasion and prognosis. CircPVT1 was increased in GC and promoted cell growth by sponging the miR-125 family (22). CircLARP4 was decreased and inhibited proliferation and invasion in GC cells by sponging miR-424 and upregulating LATS1 gene, whose expression is correlated with the pathological stage as well as unfavorable prognosis. Thus circLARP4 could act as a novel tumor suppressive factor in GC (25). CircRNA_100269 was found decreased in GC cells and inhibited the cell proliferation. Furthermore, miR-630 was verified a directly interacting with circRNA_100269 and could suppress its function in GC cells (26). CircYAP1 was decreased the overexpression of which inhibited cell proliferation as well as invasion in GC tissues by sponging miR-367-5p to inhibit p27 ^*Kip1*^ expression and GC progression (27). Meltzer’s group identified the overexpression of miR-21 in GC samples based on miR sequence data from 446 GC and 45 normal samples within The Cancer Genome Atlas (TCGA). Based on miR-21 they designed a synthetic circRNA containing five repeated miR-21 binding sites and revealed the role of the synthetic circRNA in suppressing GC growth by sponging miR-21 (51). Has_circ_0001461 was verified as decreased in GC tissues whose overexpression inhibited GC cell proliferation, migration and invasion by sponging miR-548g and upregulating RUNX1 expression in GC cells (31). CircRBMS3 was increased in GC tissues, and its expression was closely related to advanced TNM stage and lymph-node metastasis. CircRBMS3 could promote gastric cancer cells proliferation and invasion by sponging miR-153 thereby upregulating SNAI1 expression (34). CircPDSS1 was increased in GC tissues and promoted GC cell cycle and inhibited apoptosis by sponging miR-186-5p and upregulating NEK2 expression (35). Hsa_circ_0000234 was decreased in GC tissues and regulated VAMP3 expression by sponging miR-142-3p in the development of GC (36). CircPSMC3 was decreased in GC tissues which was correlated with higher TNM stage and shorter overall survival. CircPSMC3 overexpression suppressed the tumorigenesis of GC by competitively binding endogenous miR-296-5p to regulate the PTEN expression (37). CircNF1 was increased in GC tissues and promotes cell proliferation by targeting miR-16, thereby decreasing the expression of its downstream target MAP7 and AKT3 (38). Ba’s group identified an exosomal circRNA named ciRS-133, which was delivered into preadipocytes thereby promoting the white adipose tissue (WAT) browning in GC patients by suppressing miR-133 and activating PRDM16 (39). CircDLST was elevated in GC tissues and promotes the tumorigenesis and metastasis by sponging miR-502-5p thereby activating the miR-502-5p mediated NRAS/MEK1/ERK1/2 signaling (40). CircCACTIN expression was highly increased in GC tissues which promoted GC cells migration, invasion and EMT by sponging miRNA-331-3p and regulating mRNA expression of TGFBR1 (41). CircNRIP1 promotes tumorigenesis in GC by sponging miR-149-5p to downregulate AKT1. Moreover, circNRIP1 promotes tumor metastasis by transmitting exosomal communication among GC cells (42).

We realized that the functional study of circRNAs in GC is limited in their role as miRNA sponges in GC cell proliferation, invasion and prognosis. However, this may be the very first step to understanding the circRNAs functions in cancers. Some of the circRNAs which have RBPs, binding sites, or enzymes may function as protein sponges or protein scaffolds while their exact roles in cancer have not been explored (52, 53).

#### CircRNA Serves as Diagnostic Biomarkers in GC

Circular RNAs feature in abundance, structurally conserved and stable, which exhibits high tissue-specific expression (54). The high structural stability makes circRNAs resistive to ribonucleases and enables them to be detected in human blood, saliva, and gastric fluid (44, 55, 56). These characteristics increase the potential of circRNAs to become diagnostic biomarkers in GC. Additionally, circRNAs significantly correlated with distal metastasis, tumor node metastasis (TNM) stage, gender, and age in GC, which make them suitable as a cancer biomarkers (57). In recent years, plenty of studies have verified circRNAs regarding their clinical significance and the roles in GC. Their clinical relevance with GC diagnosis was summarized below.

Hsa_circ_0000190 exhibits a decreased level in GC tissues and GC patients’ plasma samples, the expressions of which are correlated with tumor diameter, lymphatic metastasis, distal metastasis, and TNM stage. The AUC in tissues and plasma was better than CEA and CA19-9 as GC biomarkers which were reached to 0.75 and 0.60, respectively (58). Hsa_circ_0000745 was decreased in GC tissues and plasma samples from patients with GC. Hsa_circ_0000745 expression in GC tissues and plasma correlated with tumor differentiation and TNM, respectively. The AUC of hsa_circ_0000745 in plasma was 0.683 and the combination of plasma hsa_circ_0000745 level and CEA level increased the AUC to 0.775, indicating hsa_circ_0000745 could be a good GC diagnosis biomarker (59). Hsa_circ_0001017 and hsa_circ_0061276 are decreased in GC tissues whose levels were significantly correlated with distal metastasis. Combinative use of above two circRNAs increases the AUG to 0.966 with 95.5% sensitivity and 95.7% specificity, suggesting their suitability as potential biomarkers in GC diagnosis (47). CircPVRL3 was decreased in GC tissues with 90.3% sensitivity and 56.4% specificity. The potential diagnostic value of the circPVLR3 was analyzed with AUC of 0.7626. Furthermore, the AUC of circPVRL3 expression varied in different TNM stages, creating the possibility for circPVLR3 to become a prognostic biomarker (60). The circSMARCA5 level was decreased in GC tissues and cell lines. CircSMARCA5 upregulation inhibited GC cells proliferation, migration and invasion, inversely, circSMARCA5 knockdown promoted GC progression. The AUC of circSMARCA5 was 0.806, indicating that the circRNA could function as a potential diagnosis biomarker (61). Hsa_circ_0000467 was significantly increased in GC tissue and in GC cell lines as well as in the plasma samples from GC patients. The AUC of hsa_circ_0000467 to discriminate GC patients and healthy individuals was 0.790, suggesting that hsa_circ_0000467 might be a potential diagnosis biomarker in GC. Moreover, hsa_circ_0000467 might act as a prognosis biomarker due to the close association between hsa_circ_0000467 and TNM stage (62). CircPSMC3 was decreased in GC tissues, GC patients’ plasmas and GC cell lines. Down-expression of circPSMC3 was negatively associated with TNM stage and lymphatic metastasis with the AUC up to 0.9326 along with the 85.85% sensitivity and 95.24% specificity, indicating that circPSMC3 is a potential biomarker with a high diagnosis value (37). Circ-DCAF6 was found increased in human GC tissues compared to adjacent non-tumor samples. The high circ-DCAF6 expression was evidently correlated with depth of invasion, lymph node invasion and TNM stages but was not associated with gender, age, tumor size and differentiation grade. At a prognosis level, the positive expression of circ-DCAF6 is highly correlated with GC patient overall survival. Therefore, circ-DCAF6 may act as a useful biomarker for GC (63).

## CircRNA in Cancer Therapy Resistance

As illustrated above, gene expression profiles screening implicated the possible role of circRNAs in GC progression, and the deregulation of circRNAs that influenced the GC cell proliferation, migration and invasion signaling, furthermore, correlated with the occurrence and development of GC mean circRNAs could become a potential diagnosis and prognosis biomarker for GC. In this manner, circRNAs may be used as a therapeutic target in GC treatment in the near future. Although we did not find any clinical reports regarding circRNAs treatment at the time of writing, accumulated evidences have demonstrated significant clinical relevance of circRNAs in cancer therapy. Here we will summarize the current status of circRNAs in cancer therapy resistance (Table 2).

**TABLE 2:** CircRNA in cancer therapy resistance.

CircRNAs	Cancer	Deregulation	Gene affected	Associated cell process	References
circPVT1	Osteosarcoma	Increased	ABCB1	Resistance to doxorubicin and cisplatin	(65)
circ_0001258	Osteosarcoma	Decreased	miR-744-3p/GSTM2		(75)
circ_0000285	Bladder cancer	Decreased		Prognosis and cisplatin resistance	(69)
circ_0025202	Breast cancer	Decreased	miR-182-5p/FOXO3a	Sensitivity to tamoxifen	(71)
circRNA-007874	Breast cancer	Increased	TRAF4/Eg5	Reverse monastrol resistance	(76)
circ_0004015	NSCLC	Increased	miR-1183/PDPK1	Promotes TKI drug resistance	(77)
circ_0004870	CRPC	Decreased	RBM39	Resistance to enzalutamide	(70)
circ_100053	Chronic myeloid leukemia	Increased		Resistance to imatinib	(78)
circBA9.3	Chronic myeloid leukemia	Increased	c-ABL1/BCR-ABL1	Resistance against TKI therapy	(72)
circPAN3	Acute myeloid leukemia	Increased	miR-153-5p/miR-183-5p-XIAP	Resistance to doxorubicin	(73)
hg19_circ_0005033	LSCC	Increased	miR-4521	Resistance to chemotherapy	(79)
hsa_circ_0000199	Gastric cancer	Increased	miR-198/PIK3R1	Resistance to CDDP	(33)
hsa_circ_0081143	Gastric cancer	Increased	miR-646/CDK6	Resistance to CDDP resistance	(74)

*CRPC, castration-resistant prostate cancer; NSCLC, non-small cell lung cancer; LSCC, laryngeal squamous cell carcinoma.*

First circRNA expression profiles based on microarray analysis using chemoradiation-resistant colorectal cancer (CRC) cells identified 71 differentially expressed circRNAs and the most modulated circRNAs were involved in signaling pathways associated with CRC development, which provided a useful database for further understanding chemoradiation resistance and the role of circRNAs in chemoradiation-resistant CRC development (64). CircPVT1, as mentioned above as a tumor promoter in GC, was found positively expressed in osteosarcoma (OS)tissues, serums as well as chemo-resistant cell lines. CircPVT1 knockdon weakens the resistance to doxorubicin and cisplatin of OS cells by decreasing ABCB1 gene expression (65). Differentially expressed circRNAs were demonstrated in Gemcitabine resistant pancreatic ductal adenocarcinoma (PDAC) cell lines and the two most increased circRNAs in patients with Gemcitabine resistance compared to the non-resistant patients were identified. Two circRNAs knockdown keep the sensitivity of PANC-1-GR cells to Gemcitabine treatment, however, overexpression of them increased the resistance (66). Screening of differentially expressed circRNA profiles in taxol-resistant and AZD9291-resistant non-small cell lung cancer (NSCLC) cells provides a database for further understanding the drug resistance in NSCLC (67, 68). Hsa_circ_0000285 was significantly reduced in bladder cancer tissues and serum and had lower expression level in cisplatin-resistant bladder cancer patients. And the association of hsa_circ_0000285 with tumor size, differentiation, lymph node metastasis, distant metastasis and TNM stage was analyzed (69). Hsa_circ_0004870 was identified of its significance in enzalutamide resistance development in castration-resistant prostate cancer (CRPC) through association with RBM39 gene (70). Hsa_circ_0025202 was significantly decreased in tamoxifen-resistant breast cancer and regulated the miR-182-5p/FOXO3a axis to achieve tamoxifen sensitivity and tumor progression (71). CircBA9.3 promoted the proliferation and inhibited apoptosis of CML cells, and was validated as increased in patients with TKI resistance, which was positively correlated with BCR-ABL1 expression (72). CircPAN3 regulated doxorubicin (ADM) resistance in acute myeloid leukemia by regulating the miR-153-5p/miR-183-5p-XIAP signaling (73). At the time of writing, two reports had demonstrated the role of circRNAs in GC resistance and the molecular mechanisms were explored. Hsa_circ_0000199 was increased in CDDP-resistant GC tissues and cells and was proved regulating the expression of PIK3R1, activating the PI3K/AKT signaling pathway and targeting miR-198 to facilitate the resistance of CDDP in GC cells (33). Hsa_circ_0081143 was increased in GC tissues and inhibition of hsa_circ_0081143 induced the GC cells sensitivity to CDDP *in vitro* and through increasing miR-646 expression to downregulate CDK6 expression *in vivo* (74). These results indicated the possibility of circRNAs to serve as potential biomarkers and therapeutic targets for various cancer types.

## Future Perspectives

**FIGURE 2:**
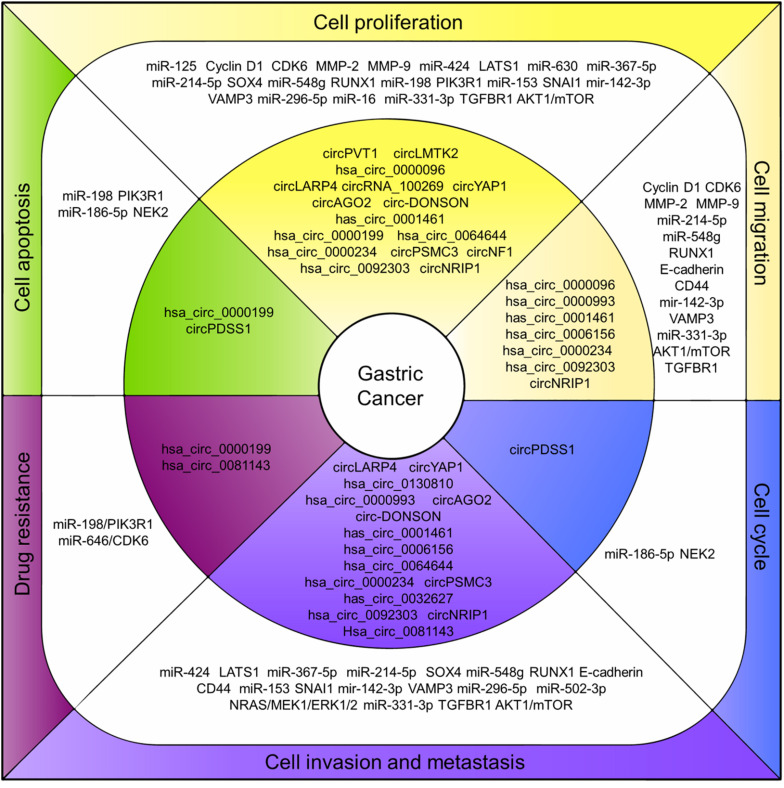
CircRNAs associated with human GC. CircRNAs associated with human GC. Deregulation of circRNAs **(inner layer)** regulates endogenous miRNAs and related proteins/pathways **(middle layer)** that ultimately lead to associated cell processes in GC **(outer layer)**.

Even though the studies focusing on the roles of circRNAs in cancers are just beginning, researchers obviously have good prospects regarding the circRNAs functioning as potential therapeutic targets for cancer treatment. As in GC, a growing number of circRNAs were characterized pathologically associated with GC and the mechanisms in regulating GC cell proliferation, invasion and prognosis were studied (Figure 2). The circRNAs are structurally stable, differentially expressed with tissue-and disease-specificity in GC making them a potential diagnosis biomarker. However, the study regarding their biogenesis, cellular location, function, and the regulation mechanism for most of the circRNAs remains insufficient.

More attention was paid to the relationship of endogenous circRNAsin GC occurrence and development in recent studies. The functional efficacy of synthetic circRNA was first reported in decreasing GC cell proliferation, suppressing the miR-21 regulatory activity in posttranscriptional levels and increasing miR-21 downstream proteins expression. In future studies, the role of the synthetic circRNA in inhibiting tumor growth and the toxicity in an animal model should be evaluated. Furthermore, the synthetic circRNA can be easily synthesized and designed thereby applied to target other cancer types or diseases and can be tested in clinical practices (51). Exosomes have been reported to be involved in almost every process of cancer (80, 81), and cell derived exosomes were used as cancer drug delivery systems that obviously enhanced drug cytotoxicity in cancer treatment (82, 83). Recent study reported that circulating exosomal miRNAs in adipose tissue was able to regulate gene expression in distant tissues, implying exosomes are important mediators in signal transduction (84). The cancer-secreted circRNAs were first demonstrated in GC that the exosomal ciRS-133 can be transported into preadipocytes and promoted white adipose tissue browning. In addition, exosome-delivered ciRS-133 aggravated tumor cachexia, of which the underlying mechanism required further study (39). The studies with new points of view not only provided a new horizon toward the possible roles of circRNAs in GC progression but also offer new perspectives for using circRNAs as a novel tool in GC treatment.

It is beyond doubt that the research of circRNAs is still in its beginning, continued work should be carried until the practical application of circRNAs for cancer treatment is better understood. Most screening studies of circRNAs mentioned above were performed using RNA seq on a limited number of GC tissues and healthy controls. Although most differently expressed circRNAs in certain cancer types can be validated using more than one prediction algorithm and RT-qPCR, the expression level of these circRNAs in other cancer types has never been evaluated. Therefore, it hampers the application of singular circRNAs as specific biomarker for a certain cancer type. So far, the studies of mechanisms of certain circRNA in cancer progression were emphasized on its regulatory function as a miRNA sponge. This is somewhat biased because we know very little about what, in practice, causes the deregulation of circRNAs in cancer at the time of writing. There is speculation that the high dysregulation may be partially due to the gene changes genetically and/or epigenetically involved in their biogenesis (85), as of yet, this is still a question that remains to be answered. Moreover, the involved miRNA might target hundreds of genes thus the regulatory network from circRNA to miRNA and mRNA must be complicated and might influence multiple cancer related signaling pathway. At the time of writing, we know almost nothing about circRNAs-targeted therapy even if a few reports have highlighted the circRNAs as master-regulators of drug resistance in several cancer types (86). Continued work should be carried out to reveal the biogenesis, regulatory functions and molecular mechanisms both pathologically and physiologically.

## Conclusion

In conclusion, circRNA research is at the beginning of its route to clinical practices. A growing number of new circRNAs have been identified that were deregulated in GC tissues, plasma and cell lines, and the deregulation was closely correlated with GC cell proliferation, apoptosis, invasion and metastasis by mediating endogenous miRNA/target genes signaling pathway. The stability and its tissue- and disease-specificity of circRNAs make them novel biomarkers and potential therapeutic targets in GC diagnosis and treatment. Moreover, circRNAs associated with drug resistance were validated in GC and other cancer types although the research is still in its infancy. In the future, the physiological aspects of circRNAs regarding the biogenesis, cellular location and functions need to be determined and their regulatory networks in GC and other diseases remain to be extensively and systemically investigated.

## Author Contributions

WW and QY designed the concept and draft the manuscript. TZ and JY analyzed the data in the references and revised the language. All authors contributed to the article and approved the submitted version.

## Conflict of Interest

The authors declare that the research was conducted in the absence of any commercial or financial relationships that could be construed as a potential conflict of interest.
